# Biographical ruptures by the COVID-19 pandemic on adolescent and young trans men and transmasculine people: demands for nursing*

**DOI:** 10.1590/1518-8345.6243.3753

**Published:** 2022-11-07

**Authors:** Anderson Reis de Sousa, Felipe Aliro Machuca-Contreras, Andréia Vanessa Carneiro de Morais, Ranna Danielle Doria de Araújo, Glauber Weder dos Santos Silva, Climene Laura de Camargo, Jeane Freitas de Oliveira, Evanilda Souza de Santana Carvalho

**Affiliations:** 1 Universidade Federal da Bahia, Escola de Enfermagem, Salvador, BA, Brazil.; 3 Universidad Autonoma de Chile, Departamento de Enfermagem, Santiago, Chile.; 4 Secretaria de Estado da Saúde Pública do Rio Grande do Norte, Natal, RN, Brazil.; 5 Universidade Estadual de Feira de Santana, Departamento de Saúde, Feira de Santana, BA, Brazil.

**Keywords:** Transsexualism, Sexual and Gender Minorities, Men’s Health, Adolescent Health, Health Vulnerability, Nursing Care

## Abstract

**Objective:**

to understand the biographical ruptures caused by the COVID-19 pandemic on adolescent and young trans men and transmasculine people in the Brazilian context.

**Method:**

qualitative study - multicenter, online survey. A total of 97 self-identified trans men and 22 transmasculine people participated and completed a semi-structured form in two stages. The data was subjected to Reflective Thematic Content Analysis. The interpretation was made on a sociological basis, based on the concept of biographical rupture.

**Results:**

five categories were derived: interruption of hormonization, surgeries and specialized follow-up; discomforts caused by the rupture of masculine characteristics, self-image, self-perception, and identity; vulnerability from the losses of family members and significant people, employment, and weakening of support networks; emergence of psycho-emotional problems, such as loss of meaning in life; demands for nursing care and valuing the life of transmasculine adolescents and young men in post-pandemic times.

**Conclusion:**

the biographical ruptures caused by the pandemic threatened the identities of trans and transmasculine people of adolescents and youth, degraded and interrupted biographies, leading them to the loss of meaning in life. Nursing professionals can be strategic and essential in overcoming threats by intervening early.

## Introduction

During the COVID-19 pandemic, specific groups, such as trans population, have experienced multiple life and health impacts^1^ ^-^ ^3^ ^)^ that have not yet been considered in coping strategies. A Brazilian study estimated that approximately three million (1.9%) trans people live in the country and are, on average, younger than the cisgender population (32.8±14.2 years)^4^.

The voices of trans men - men who were assigned the female gender at birth - and of transmasculine people - people whose gender identity is male, but who do not necessarily identify as men, which involves a masculinity dimension, in this case, transmasculinities^5^ ^-^ ^6^ - have been silenced in decision-making and the subjects invisibilized in the construction of public policies^7^. This structural and systemic violence has a patriarchal and gender-normative component, which has historically silenced vulnerable groups, a phenomenon that can be explained from the concept of biographical ruptures (BR)^8^ ^-^ ^10^.

Faced with biographical ruptures, people develop the processing of reality through three interrelated and distinct characteristics. Firstly, the onset of the illness experience, which we will define as the causal condition of BR (which can be an illness or threat to integrity), implies the cracking of assumptions and behaviors incorporated into everyday life, which leads the person to conceive life as a chaotic set of disconnected events^8^. Secondly, one experiences “rethinking one’s biography and self-concept.” At this point, people are urged to consciously reflect on what they thought about their own futures and how they will have to manage the interruptions of those futures^8^. Finally, the mobilization of new resources to deal with the chaos arising from BR is observed^11^.

The scientific literature suggests the aggravation of health problems, precariousness of care, and overlapping barriers to comprehensive health care, which reiterates the justification for this study^12^ ^-^ ^13^. Also, considering the fact that trans people have different health needs and that existing health programs cannot address multifactorial problems and provide coverage for the trans population, the scenario becomes even more worrisome^14^ ^-^ ^15^. Thus, this theoretical movement of practical and epistemological disobedience should prompt special attention to the adolescent life cycle^16^ and signals the existence of a global health problem that is a priority on the public agenda. This justifies the usefulness and relevance of this study, as well as the gap in the production of scientific knowledge on this subject.

We start from the assumption that from the COVID-19 pandemic all humans experienced a biographical rupture or threat, and therefore we are interested in understanding the singularities of this phenomenon for adolescents and young people who were already experiencing a daily gender-affirming life. In this sense, this research was guided by the question: how did adolescent and young trans men and transmasculine people experience the biographical ruptures arising from the COVID-19 pandemic in Brazil? This study aimed to understand the biographical ruptures caused by the COVID-19 pandemic on adolescent and young trans men and transmasculine people in Brazil.

## Method

### Study design

This is a qualitative, sociological study^17^, structured based on the recommendations of the Consolidated Criteria for Reporting Qualitative Studies *-* COREQ. An online^18^, national multicenter survey entitled: “Analysis of the health impacts of the COVID-19 pandemic on trans men and transmasculine people” was conducted.

### Data collection site

The research addressed all regions of Brazil, using consecutive chain sampling, snowball sampling^19^. Participants were recruited on digital social media: Facebook, Instagram, Twitter, Scruff, Grindr, Tinder, in groups of trans men and transmasculine people, and/or transgender and/or lesbian, gay, bisexual, travesti, transsexual, queer, intersex, asexual, or those with experiences of gender variability, represented by the “+” symbol (LGBTQIA+) in WhatsApp and Telegram apps^20^.

To determine the zero wave of recruitment, initially ten participants, trans men and transmasculine people, were included as “seeds” ‒ a term adopted to designate the participants who initiated the reference chain sampling, chosen non-randomly^19^. They were encouraged to invite new participants, which made it possible to track the recruitment of five major seeds, one from each region of the country, as well as the respective chains of new informants, the “seed children” ‒ individuals recruited by the seeds-and resulted in representation from 17 states^21^.

### Instruments used for data collection

**Figure 1:**
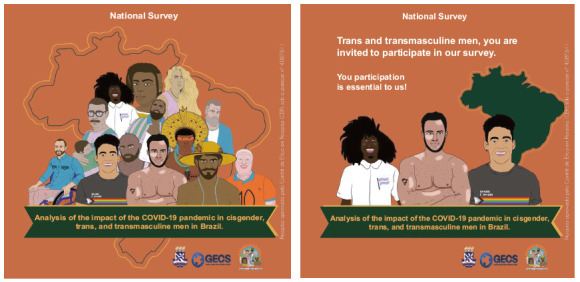
Themed cards for research dissemination. Salvador, BA, Brazil, 2022

**Figure 2:**
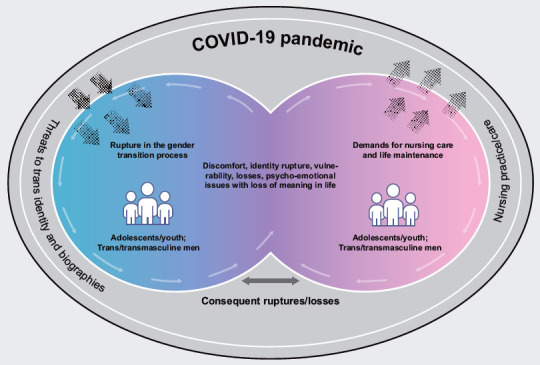
Explanatory model of the experience of adolescent and young trans men and transmasculine people in the COVID-19 pandemic in Brazil in the face of biographical ruptures. Salvador, BA, Brazil, 2022

Themed cards with virtual avatars were used to communicate with the participants, as illustrated in Figures 1 and 2. Moreover, the dissemination on social networks occurred by profiles and pages created exclusively for the project, namely: @cuidadoasaudedehomens, and the hashtags: *#pesquisasaudehomenstransnapandemia; #pesquisasaudasaudepessoastransmasculinasnapandemia; #saudedepessoastransmasculinaspandemia; #saúdehomenstranspandemia,* and of access to the participants: *#homenstrans*; *#pessoatransmasculina*; *#transmasculinos*; *#garotostrans* and *#adolescentestrans*. 

### Participants

A total of 97 trans men and 22 self-identified transmasculine people who met the inclusion criteria participated in the study: being 18 years or older, dwelling in Brazil, being in the country during the COVID-19 pandemic, self-affirming/recognizing themselves with male gender identity. People who were passing through Brazil were excluded from the study ‒ international travelers and newcomers to the country, in an immigration or refugee situation. The definition of adolescent/youth in this study was based on the consensus of the World Health Organization (WHO) and, for ethical reasons, participants aged 18 years or older were selected due to the impossibility of contacting the guardians of minors remotely^22^.

### Data collection

Data were collected in three stages. The first was conducted from March to December 2021, using a self-applied online semi-structured form, hosted on the Google Forms web software application, taking from 20 to 30 minutes. This choice was due to the wide dissemination of the tool in Brazil, besides being free, easy to access, allowing the creation and editing of online surveys, with collaboration of different authors in real time, creating personalized design/themes, submitting the collected information automatically to a spreadsheet, and making available Uniform Resource Locator (URL), under the offer of upload file for access by the participants^23^ ^-^ ^24^. The software has features for intelligent response validation, which allows text entry detection and correction. The application also adopts security and data protection measures, in convergence with the General Law of Data Protection (LGPD) in force in the country^25^.

The form integrated questions related to socio-identity, economic, labor, and health characterization data, in addition to the following open questions: after one year of the COVID-19 pandemic in Brazil, have you experienced anything important and representative in relation to your health that you wish to tell us about? Has the COVID-19 pandemic had any impact on health and health care? Describe what happened.

In the second stage, in February 2022, remote individual interviews were conducted with the same respondents (20 randomly chosen participants), via Google Meet and WhatsApp video calls and audio messages, guided by a script consisting of open-ended questions. The interviews occurred in a single meeting. They were previously scheduled, upon authorization and availability of the participants, in order to answer the guiding question: tell us more about the health impacts experienced after the pandemic?

In possession of this data set, the research team: (1) extracted the data from the spreadsheet generated by the Google Forms; (2) checking the data for integrity, duplication, and incompleteness; (3) organizing and systematizing them in customized files using identification codes; (4) transcribing and linguistically correcting the interview data; (5) preparing the *corpus*; (6) transferring data to NVivo12 software, and (7) analyzing data^26^.

In the third stage, which took place in March 2022, the categories and subcategories found in the first stage were validated with the participants; there were also debates on strategies for health care and coping with the adversities evidenced. Thus, an invitation and an online form were sent, answered by 10 participants, containing the description of categories and subcategories obtained in the initial analysis. In this step, participants were asked: what are the coping and health care strategies for trans men and transmasculine people? Would you point it as important to overcome the problem presented in the category? This stage followed the same analysis steps and saturation criteria described in the first and second stages. Theoretical saturation of the data was considered from the empirical theoretical density presented in the findings^26^.

### Data treatment and analysis

All empirical material was subjected to a Reflexive Thematic Content Analysis in all three stages of the research. To this end, several cyclical moments of data analysis were performed, in a spiral fashion, as a way to ensure greater theoretical abstraction and reflexivity. The in-depth reading was carried out, along with the constitution of the *corpus* of analysis, followed by the attribution of labels, the adjustment of data homogeneity, the formulation of empirical indicators of analysis, the ordering, the naming of codes and the derivation of categories, subcategories and respective themes^27^ ^-^ ^29^. Finally, the research team internally validated the codes, through peer consensus, as criteria for achieving the quality of the analysis, and submitted the categories to the participants for corroboration^30^.

After the participants validated the categories and subcategories obtained in the steps, the research team reconfigured their presentation based on suggested adjustments. Furthermore, the questions asked in the third stage brought out two new categories. Then, the findings were interpreted from the theoretical concept of BR. Although this concept was inaugurated by medical sociology and considered the experience of illness as a referent, in this study we adopted the more extended concept that BR involves a rupture/fissure over the individual’s ability to enact an embodied orientation toward the world. In this sense, it does not result from illness, but from the ways in which processes that threaten integrity affect a person’s abilities to engage with everyday life^8^ ^-^ ^11^. Thus, the concept has proven useful for analyzing the investigated phenomenon in the face of the set of threats caused by the COVID-19 pandemic, such as transphobia.

### Ethical aspects

This study followed the ethical recommendations in all stages and met the Regulation 466 of 2012 of the National Health Council^31^, the guidelines for research in the virtual environment, the care of surveillance and protection of the data generated: use of passwords, codes and zipped folders, no use of collective emails, no storage of data in clouds, preservation of anonymity with identification of participants by means of the initial H and ordinal number, for example, H01, H02, H03…^32^ ^-^ ^33^. In addition, an informed consent form was used in the imaging modality, and its copy made available by email or other modality of the participant’s choice.

## Results

Most participants self-identified as trans men (97), followed by transmasculine people (22); heterosexual sexual orientation, Black (52 Black and 47 Brown), aged from 18 to 24 years old, single, with complete secondary education. Regarding salary income, the participants were classified as having no income and earning up to 2,900 BRL, which decreased significantly in the pandemic. They worked in informal activities (self-employed, without a signed contract, student with income).

As for the health situation, they pointed out problems such as: anxiety, asthma, respiratory and dermatological/cutaneous allergies, depression, diabetes mellitus, dyslipidemia, epilepsy, gastritis, glaucoma, arterial hypertension, human immunodeficiency virus (HIV), hyper and hypothyroidism, medullary hypoplasia, obesity, polycystic ovaries, bipolar disorder, panic disorder. They used exclusively the Unified Health System (SUS), evaluated their mental health situation as bad, followed by very bad, and considered their spiritual, physical, and sexual health as regular. Most were not diagnosed with COVID-19, however 18 participants said they experienced complications from COVID-19 and six had reinfection and were vaccinated against the disease. Finally, 47 participants reported having lost a close person to COVID-19.

From the content analysis, five thematic categories emerged, presented below:

### Central theme - Ruptures in the process of gender affirmation threaten transmasculine identities and biographies

#### Category 1 - Hormonization interruption, surgeries and specialized follow-up

[…] *I could not perform routine tests, nor find hormones available in pharmacies.* (H11); […] *my appointments were all canceled, I had to postpone my breast removal surgery.* (H43); […]*the paralysis of care in the trans outpatient clinics harmed my hormonal transition.* (H58); […] the *production of testosterone decreased in the pandemic, brought impacts to harmonization, there was no policy to offer the hormones, by the government, which denies the trans existence, does not care about our health.* (H72); […] *I had to interrupt the transition and wait for the return of consultations and follow-up in the trans outpatient clinic.* (H70); […] *I postponed the mastectomy*; *gender reassignment, through masculinizing mastectomy and hysterectomy was something very important, but stopped with the pandemic*. (H119).

#### Category 2 - Discomfort caused by the breakdown of masculine characteristics, self-image, self-perception, and identity

[*…*] *I had to withdraw from the follow-ups and this damaged the way I saw and perceived myself as a man.* (H10); […] *it has been very difficult to make appointments in the Unified Health System (SUS), and without the hormones I cannot achieve male passability.* (H11); […] *I have only been left to search for emergency services, but they do not solve the demands of transition.* (H28); […] *the difficulty in performing diagnostic exams and evaluation prevents me from keeping the hormone transition. With that, I don’t reach the masculine characteristics.*(H36); […] *I already have more than 10 years in hormonization, I am in an advanced process of transition. This means that the worries about maintaining the masculine characteristic and/or a standard of appearance, through testosterone applications, diminishes a little, does not become absent, because my body asks for hormone maintenance. It is an indispensable demand, as is the insertion of a IUD.* (H119).

#### Category 3 - Vulnerability from the loss of family and significant others, employment, and weakening of support networks

[…] *I lost my mother to COVID-19, which left me very vulnerable emotionally.* (H1); […] *I started to develop social phobia*. (H13); […] *I lost friends to COVID-19, some were trans and it had a big impact on my social interaction and mental health.* (H17); […] *the loss of important people left me helpless.* (H40); […] *I live alone, and the pandemic left me in “the low” because of loneliness, isolation and absence of company.* (H52). […] *I lost my job, my health problems increased.* (H35); *[…] everything became more difficult financially. (*H27 ); […] *difficulties increased to keep me in school, due to lack of income.* (H31); […] *I lost my job, I had no fixed income, I was prevented from studying.* (H32); *I had difficulty feeding myself and buying gas for cooking.* (H46); […] *made it difficult to have access to alcohol gel, disposable mask.* (H92)*.*

#### Category 4 - Emergence of psycho-emotional problems with loss of meaning in life

[…] *I attempted suicide.* (H05); […] *I discovered that I have bipolar disorder and worsening depression.* (H27); […] *I experienced many episodes of suicidal ideation.* (H41); […] *it seemed that my psyche was no longer present or functioning. Suicidal thoughts were constant in the pandemic. I thought about ending my life.* (H46); […] *I stopped having interest for life, gained 15 kilos and tried to kill myself.* (H40); […] *during the pandemic I received the medical report that borderline personality and bipolar disorders.*(H41); […] *anxiety and depression increased so much that I attempted suicide.* (H43); […] *unbearable suffering and anguish, so intense that I even thought of suicide.* (H47); […] *my days are almost always sad and with psychosis, aggravated by isolation and intense work in the pandemic. I no longer feel the desire to live*. (H54); *I tried to take my life by taking medication, because I couldn’t stand it anymore.* (H65); […] *I was diagnosed with depression and generalized anxiety disorder and panic syndrome.* (H94)*.*

#### Category 5 - Demands for nursing care and valuing the lives of adolescent and young trans men and transmasculine people in post-pandemic times

[…] *nursing care for trans people needs to be expanded to reduce mental health harms.* (H10); […] *the creation of virtual educational actions for trans men can provide information related to care of the physical body, mobility, hygiene and immunity, important in times of social isolation.* (H11); […] *creating a specific care line for trans men with post-COVID-19 sequelae would be a great contribution of nursing.* (H27); […] *can develop health programs to face gender dysphorias.* (H37); […] *intervene in front of the most prevalent psychiatric aggravations.* (H54); […] *nurses can invest in the construction and dissemination of educational bulletins about mental health care for trans men.* (H65);[…] *the use of hormones should be worked on by nursing professionals, seeking to create protocols for handling, guidance on domestic use, types to be used, interactions with other drugs, sites of application, necessary tests, evaluation of metabolic rates, relationship with gynecological demands, such as lubrication and/or vaginal dryness.* (H94); […] *nurses could create educational psychosocial support groups, in person or virtually and free of charge, in order to improve the affective bonds between professionals and trans men and transmasculine people.* (H112).

From the analytical process, an explanatory model of the experience of adolescent and young trans men and transmasculine people in the COVID-19 pandemic in Brazil in the face of biographical ruptures was elaborated (Figure 2). The model highlights biographical ruptures, especially those related to the body and gender (assigned gender identification), the interruption of social reassignment, access to body adaptation technologies, and the maintenance of mental health, compromised by the exacerbation of problems already experienced and the emergence of new psycho-emotional disorders. In addition, there is evidence of increased barriers in accessing health services and technologies, in achieving and maintaining income, as well as impoverishment and employment, intellectual, and labor vulnerability.

## Discussion

This study revealed that biographical ruptures added to the syndemic effects of the pandemic compromised the processes of gender transition, having negative repercussions on health, quality of life, psychological and social well-being, self-care and health practices, and the quality of care offered in the network of services available in Brazil. The participants in this study cast a sharp look at the services and care, and proposed measures to overcome this scenario.

The biographical ruptures experienced by adolescent and young trans men and transmasculine people are evidenced by changes in the process of gender affirmation, which involves hormonization, access to body-fitting technologies, sexual reassignment, and specialized follow-up. More than that, they experienced scratches on self-image, self-concept, identification, discomforts generated by the discontinuity of care for the maintenance of masculine characteristics attributed to the body/gender/image, as well as the production of threats to transgender male identities and biographies in adolescence and youth, which contributed to the manifestation of psychic suffering.

The nursing team’s sensitive and technically qualified performance and the integration of the multiprofessional health team and of transdisciplinary areas are essential for the subjects to achieve their individual goals related to appearance and “passability” during this vital cycle^4^. In addition, such actions may involve aspects such as the choice of a name, management of conflicting situations such as menstruation, breast volume and the need to hide them with the use of binders, difficulty in thickening the voice and vocal adequacy, hair growth ‒ configuration of the beard ‒, application of the first doses of testosterone and even the use/employment of objects for building “penile volume” (briefs with volume/filling), use of prosthesis (packers) in daily life and during intimate relations and/or pump for clitoral growth^34^.

This study evidenced the abrupt suspension of the care processes that ensured the corporeality and identity preservation of the participants. It is noteworthy that in addition to the suspension of specialized care for those in the transition process, many people who have recently sought gender-affirming health care have had to postpone their projects^3^.

By causing discontinuities in the ways of living and in the identity trajectories expressed by individuals, the pandemic as a critical situation may be responsible for the transformation of lives and the narrative reconstruction of biographies^8^ ^-^ ^11^, which may reproduce narrative re-elaborations of the relations between future, past, and present^10^. Thus, we add the need to understand the overlapping and intersecting social markers ‒ for example, being adolescent, trans and/or transmasculine, Black, poor, homosexual ‒ that amplified the vulnerabilities in the pandemic in Latin America^35^.

Our findings indicate the complexity of deteriorating physical and psychosocial health status, signaling declining well-living and psychosocial well-being in future years. In addition to damage to corporeality and mental health directly and indirectly caused by the pandemic, participants were affected by complications from COVID-19, such as lasting symptoms from disease syndromes and/or sequelae. They also showed threats to the maintenance of trans identity in socialization spaces, such as in the school and university environment, in the world of work and formal employability, in the achievement of economic-financial profitability for subsistence in times of crisis, which added to the weakening of socio-affective, formal/institutional support networks or information, which puts the researched population at an extreme disadvantage in terms of protection, social security and access to human rights.

From these data, we reinforce the need to pay attention to the wishes and desires, often managed by the environmentalization of trans people in public spaces from the social and collective violence committed ‒ bullying, rejection, discrimination, stigmatization, stereotyped social representations^36^. Thus, the population that was already stigmatized suffered the greatest impacts, triggering illnesses, such as depression and anxiety, related to prejudice in the workplace, which generated even more insecurity and uncertainty. For trans people, decreased income has resulted in less access to routine hormone treatments and, consequently, body changes affecting their gender identity, a significant stressor^37^, as verified in this study.

Economic instability and barriers to the right to human dignity, often related to the internalization of prejudice and doubts inherent to the period of youth, cause trans adolescents and young people to blame themselves, to lose the meaning and purpose of life, to diminish their power strength, joy, and hope. Following this path, often with no way out, they attempt suicide^38^ ^-^ ^40^.

The hopelessness in the lives of trans people arises from the first expressions of the gender identity with which they identify, at as early as adolescence. For example, a study that assessed the prevalence of suicidal ideation in a northeastern trans population in Brazil noted that the sample had “come out” regarding their gender identity before the age of 18 (67.3%). In the group studied, those who came out in adolescence had a higher prevalence of suicidal ideation (36.2%)^39^. This phase of life is consonant with the beginning of expressions of violence and aggression in their lives^3^ ^,^ ^41^ ^-^ ^42^. Not responding to the wishes and norms of the families is the first major obstacle experienced by these people^43^. For these reasons, suicidal behavior becomes common in the trans community, especially during youth, associated with the vexatious and violent situations to which they are exposed, causing melancholy, egodystonia, and deep sadness^44^.

In the United States, economic inequities have been recorded in the LGBTQIA+ community. It was identified that 19% of transgender and gender-diverse people and 26% of transgender ethnic minorities and gender diverse people of color lost jobs compared to 12% of the general population^3^ ^,^ ^45^. Another investigation compared cisgender and trans people in the COVID-19 pandemic and pointed to significantly increased financial instability and life degradation in the transgender public^46^.

Decreased access to care and restricted participation in social connection groups have led to worsening mental health indicators, with increased rates of depression, anxiety, and suicide^47^. One study pointed out that for LGBTQIA+ teens, during the COVID-19 pandemic there was increased reliance on accessing anonymous forums for discussion of anxiety-provoking topics. Presumably this is because anonymous forums are perceived as safe spaces to discuss lifestyle stressors in the face of restrictions on meetings, such as school closings^48^.

The worsening of the social determinants of health during the pandemic is an alarm that signals the importance of attention to the mental health of the LGBTQIA+ population beyond the markers of sexual/reproductive health markers, which focus on care actions for this public, often reduced to the prevention and treatment of sexually transmitted infections (STI)^49^ ^-^ ^50^.

Trans adolescence in countries like Brazil, whose life expectancy for transgender people is 35 years, being the country that kills the most transgender and travesti people in the world, has been permeated by invasion of autonomy and private forum violence, experienced even in childhood in the intra-familial environment, resulting in “home evasion” and violent behavior in adulthood^50^. This troubled scenario for adolescent experience in transgenderness can lead to vulnerabilities such as prostitution and forced migration involving sex work with high-risk of HIV infection as recorded by the scientific literature on trans women in the United States^51^ and trans men in Canada^52^.

Even in the face of multiple difficulties, these results indicated that adolescents and young trans men and transmasculine people exercised self-perception and perception of health regarding the COVID-19 pandemic. Moreover, they indicated the weaknesses of the services that provide health care, which can be understood as a complaint, but also as a call for help. In face of everything they experienced, the participants indicated ways to overcome the problems generated by the pandemic, which public managers from governmental and non-governmental organizations, as well as nursing professionals, can benefit from, by knowing and then intervening.

In this way, by facing the threats to identities and the ruptures imposed by the pandemic, the propositions made can ensure mastery over the chaos and the resumption of their unfinished projects, and thus ensure a future regaining of control over their daily lives^8^ ^-^ ^11^. Balance after such ruptures will claim acceptance of the ever-changing trans body, for living implies relating to your body and putting it in continuous interaction with other bodies^49^.

Thus, specifically for the nursing field, this study pointed out several propositions that can contribute to the redefinition of nursing actions/interventions, competencies, and skills in men’s health^53^ ^-^ ^54^. This research also suggests the development of new proposals, which can happen in the field of management of the service and nursing care training and research, from the development of projects and investigations committed to improving the quality/expectancy of life of the transgender population, in addition to the technical and professional performance in clinical and educational intervention with adolescents and young trans men and transmasculine people^55^, as the protection of male mental health and the improvement of living habits and post-pandemic care^56^ ^-^ ^57^.

In this sense, the engagement of nursing professionals in the welcoming and the production of sensitive and inclusive care is urgent. In addition, one should advocate for the strengthening of already institutionalized initiatives, such as the School Health Program, the set of actions of the National Policy for the Integral Health of Lesbian, Gay, Bisexual, Travesti and Transgender People, and the National Policy for the Integral Attention to Men’s Health, the actions of the Children and Youth Courts, and the Special Reference Centers for Children and Youth, and the Specialized Reference Centers for Social Assistance. We must also defend the existence of social movements and collectives of trans and transmasculine people, whether in person or virtually.

This study has limitations, as it shows a partial picture of reality, since the multiple intersectionalities that are at play in the particular contexts open new boundaries that need to be explored. Regardless of whether the phenomenon is temporary (syndemic), there is a social disease (reinforced during the pandemic), namely transphobia, which remains a common thread in the experiences of young male trans people.

The analytical efforts presented have made it possible to bring out interdicted voices and build a space of vindication through research. This study also revealed how the COVID-19 pandemic produced degradation and discontinuity (ruptures) in the biographies of the participants, expressed in identity compromise, enabled by gender transition, leading to a loss of meaning in life. Thus, we demonstrated the impacts on the lives of young trans men and transmasculine people and contributed to the advancement of knowledge in the field by building an explanatory model that can serve as an instructional tool in nursing care.

## Conclusion

This study understands that the COVID-19 pandemic caused damages to the mental health and quality of life of the studied population, evidenced by the biographical ruptures in the process of gender transition and affirmation and sexual reassignment, as well as by the difficulty in accessing health services.

This research also indicates possibilities for nursing work in promoting healthy transgenderness in adolescence and youth, acting with strategic and essential actions in overcoming threats by intervening early. Furthermore, the study shows results that will be useful to instrumentalize public agents, health professionals, and the organized civil society in the promotion and prevention of health problems, in a collective exercise of post-pandemic resilience.
